# The Enfield Cottage Hospital Scandal

**Published:** 1919-03-22

**Authors:** 


### THE ENFIELD COTTAGE HOSPITAL SCANDAL.

**The undermentioned members of the Council of Enfield Cottage Hospital have issued the following statement to the Governors :**

Before the annual general meeting takes place on March 29 the members of the Council whose names are appended consider it desirable to submit to the governors a statement of the circumstances which have arisen since the special meeting of the governors, held on January 18, 1919.

It will be remembered that at that meeting the Council reported that, owing to a difference of opinion between the Council and the hon. medical staff on a question of procedure in arranging a conference, the members of the staff had declined to co-operate further with the Council, and had combined to violate one of the fundamental rules of the hospital by sending in patients without medical certificates. The governors present at the meeting, with one dissentient, passed the following Resolution:—

"This meeting of the governors of the Enfield Cottage Hospital is of opinion that the strict observance of the rules of the hospital (for the time being in force) by all those using, or ministering in, the hospital, is absolutely essential to the well-being of the institution and to its continued existence."

A copy of this resolution was forthwith transmitted to the hon. medical staff, but no notice was taken of it, and the medical staff continued to send in patients without medical certificates.

At a special meeting of the Council, held on February 8, to consider the position, the following communication was submitted from the hon. medical staff:—

"If the Council, at its meeting on February 8 next, agree to arrange that the Conference asked for by the hon. medical staff on October 5, 1918, be convened within the next fortnight, the hon. medical staff will immediately resume the signing of all hospital letters, etc., such Conference to consist of an equal number of the Council and the hon. medical staff."

In reply thereto the following resolution was moved by Mr. H. Bailey, and seconded by Mr. A. J. Hollington, and carried unanimously, a copy being subsequently forwarded to the medical staff:—

"The Council have had before them the resolution of the hon. medical staff, of February 7, and in reply they have to intimate to the hon. medical staff that unless and until medical certificates, in accordance with the rules, are signed for all patients now in the hospital or who may be sent in by them, the Council must decline to meet the medical staff in conference as to any alteration of the existing rules. If, however, the medical staff, before the meeting of the House Committee on Tuesday, the 11th instant, intimate to the Secretary that they are willing to sign certificates, as mentioned above, and will sign them forthwith, the Council will be prepared to appoint a Committee of their members to meet an equal number of members of the medical staff, to ascertain what alterations in the rules they desire, and to report to the Council."

No reply to this resolution has been received, and down to the present time no medical certificates have been furnished for any of the patients sent in to the hospital by the hon. medical staff, notwithstanding the resolution of the governors at the special general meeting referred to above.

The Council have given the most careful consideration to the course which should be adopted to deal with this breach of the regulations of the hospital. Several alternatives presented themselves. The medical staff could have been requested to resign, or they could have been removed from office under Rule 40. The objection to either proceeding was that, although no longer members of the hon. medical staff, they might still send in patients without certificates, and thus continue to disobey the rules.

Another alternative was to issue instructions to the Acting-Matron that no patient was to be admitted to the hospital without a medical certificate. But, whilst such instructions were in force, there might possibly be some urgent cases needing hospital treatment, the exclusion of which might be of serious consequence, and the Council were most reluctant to take any step which would close the doors of the hospital to any eligible necessitous case.

The position which has been created by the action of the medical staff is one which the Council feel themselves, under existing circumstances, unable to deal with adequately without running the risk of impairing the usefulness of the hospital.

It is essential for the well-being of the hospital that harmony and good-feeling should exist between the administrative and medical sides. Although the Council have done their utmost to work amicably with the medical staff, their efforts have been unsuccessful, and they feel it is impossible for them to continue in office whilst they are subjected to the continual opposition and animosity which have characterised the attitude of some members of the medical staff for a long time past. After giving most anxious consideration to the course they should adopt, the undersigned members of the Council have come to the conclusion that the only course open to them is to retire from the management of the hospital. They therefore take this opportunity of informing the governors that they do not intend to offer themselves for re-election at the annual general meeting. It is important, therefore, that the governors of the hospital should be prepared to elect other members of Council at that meeting, and, as nominations must be received by the Hon. Secretary not later than the 17th inst., it is hoped that the governors to whom this statement is addressed will nominate candidates who, if elected, may be able to safeguard the interests of those for whom the hospital is intended, without encountering opposition from the hon. medical staff.

| | |
|---|---|
| Henry Bailey. | H. Kenneth. |
| T. Briggs. | Florence Maitland. |
| Walter J. Crook. | J. Haworth Roberts. |
| Walter F. Cunliffe. | E. W. Sawyer. |
| Hubert Gough. | Mary Spicer. |
| W. S. Hale. | P. G. Vanderpump. |

F. W. Fletcher, *Chairman.*

F. Porter, *Hon. Treasurer.* James Penny, *Hon. Secretary.*

